# Supplementary material for: Splice-Junction-Based Mapping of Alternative Isoforms in the Human Proteome
Source: Cell Rep. Author manuscript; Available in PMC 2020 Jan 15. (PMC6961840; doi:10.1016/j.celrep.2019.11.026)
Supplement: 3 [file NIHMS1546469-supplement-3.zip › DF2/PXD006675/LeftAtrium-338-Q92626-YLYLHFNQIETLDPDSFQHLPK.pdf]

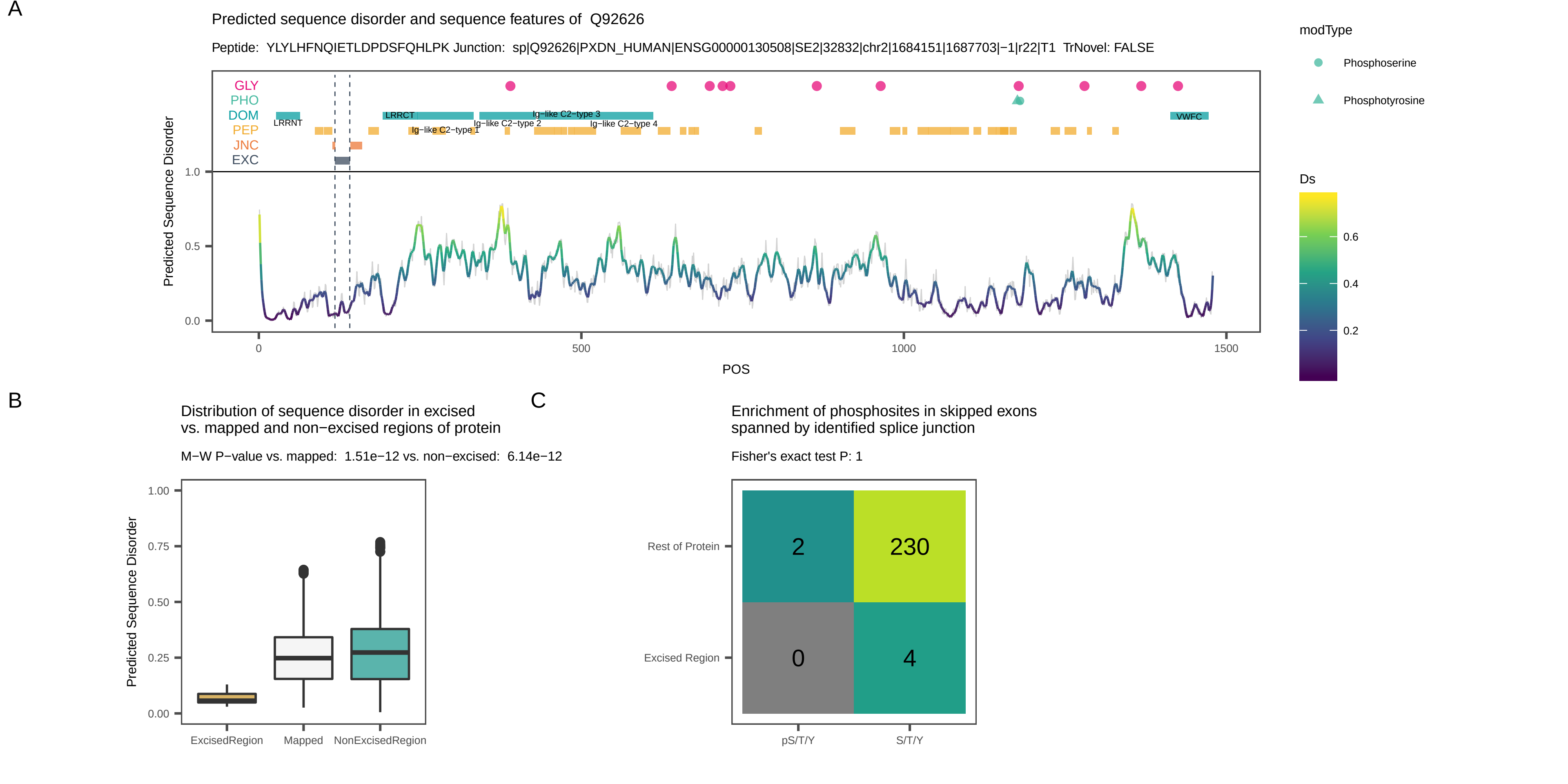
A
Predicted sequence disorder and sequence features of Q92626
Peptide: YLYLHFNQIETLDPDSFQHLPK Junction: sp|Q92626|PXDN_HUMAN|ENSG00000130508|SE2|32832|chr2|1684151|1687703|−1|r22|T1 TrNovel: FALSE
GLY
PHO
DOM
PEP
JNC
EXC
LRRNT
LRRCT
Ig−like C2−type 1
Ig−like C2−type 2
Ig−like C2−type 3
Ig−like C2−type 4
VWFC
Predicted Sequence Disorder
1.0
0.5
0.0
0
500
1000
1500
POS
modType
Phosphoserine
Phosphotyrosine
Ds
0.6
0.4
0.2
B
Distribution of sequence disorder in excised
vs. mapped and non−excised regions of protein
M−W P−value vs. mapped: 1.51e−12 vs. non−excised: 6.14e−12
Predicted Sequence Disorder
1.00
0.75
0.50
0.25
0.00
ExcisedRegion
Mapped
NonExcisedRegion
C
Enrichment of phosphosites in skipped exons
spanned by identified splice junction
Fisher's exact test P: 1
Rest of Protein
Excised Region
2
230
0
4
pS/T/Y
S/T/Y
